# Hospital board oversight of quality and safety: a stakeholder analysis exploring the role of trust and intelligence

**DOI:** 10.1186/s12913-015-0771-x

**Published:** 2015-06-16

**Authors:** Ross Millar, Tim Freeman, Russell Mannion

**Affiliations:** Health Services Management Centre, University of Birmingham, Park House, 40 Edgbaston Park Road, B15 2RT Birmingham, UK; Department of Leadership, Work and Organisation, Middlesex University Business School, 131 Williams Building, The Burroughs, Hendon, London, NW4 4BT UK

**Keywords:** Boards, Quality, Patient Safety, Trust, Intelligence

## Abstract

**Background:**

Hospital boards, those executive members charged with developing appropriate organisational strategies and cultures, have an important role to play in safeguarding the care provided by their organisation. However, recent concerns have been raised over boards’ ability to enact their duty to ensure the quality and safety of care. This paper offers critical reflection on the relationship between hospital board oversight and patient safety. In doing so it highlights new perspectives and suggestions for developing this area of study.

**Methods:**

The article draws on 10 interviews with key informants and policy actors who form part of the ‘issue network’ interested in the promotion of patient safety in the English National Health Service.

**Results:**

The interviews surfaced a series of narratives regarding hospital board oversight of patient safety. These elaborated on the role of trust and intelligence in highlighting the potential dangers and limitations of approaches to hospital board oversight which have been narrowly focused on a risk-based view of organisational performance. In response, a need to engage with the development of trust based organisational relationships is identified, in which effective board oversight is built on ‘trust’ characterised by styles of leadership and behaviours that are attentive to the needs and concerns of both staff and patients. Effective board oversight also requires the gathering and triangulating of ‘intelligence’ generated from both national and local information sources.

**Conclusions:**

We call for a re-imagination of hospital board oversight in the light of these different perspectives and articulate an emerging research agenda in this area.

## Background

Improving the quality and safety of health care services remains a key concern for policy makers and practitioners across the globe. Interest in this area continues to increase as a growing number of empirical studies document the organisational dimensions associated with high quality and safe care. Such dimensions include the importance of open channels of communication which are founded on mutual trust, strategies to enhance organisational learning, and the desirability of non-punitive approaches to adverse event reporting [1].

Hospital boards have responsibility for developing appropriate organisational strategies, incentives and cultures to support the delivery of quality and safety within their organisation. Despite this fiduciary responsibility, questions have been raised about the extent to which Boards have paid sufficient attention to quality and safety of care. In the US, the 2010 Affordable Care Act required hospital boards to take a more proactive role in strengthening their governance processes to ensure improvements in quality and efficiency [2]. In England, the high profile and well documented failures in professional practice at Mid Staffordshire hospital trust raised serious concerns about boards’ reliance on external systems of checking, verification and audit at the expense of wider considerations relating to patient care. The Francis Inquiry has provided further calls for a rethinking of Boards’ role in this regard [3,4].

A growing body of work suggests that high quality healthcare organisations are characterised by boards that show committed leadership, have an agenda that makes safety a priority and are comprised of members trained in, and knowledgeable about, the theory and practice of quality improvement methodologies [5,6]. Boards which combine a culture of ‘high trust’ across executive and non-executive members, together with robust methods of measuring and monitoring the quality and safety of patient care, are also associated with higher performance [7]. Nonetheless, Chambers [7] argues that the features of ‘high performing’ boards remain open to debate with only limited evidence on, and agreement about, the characteristics of an “ideal” Board. This view is supported by a recent review by Millar et al. [6] who suggest that our understanding of hospital board oversight in relation to quality and patient safety remains theoretically and methodologically underdeveloped.

The aim of this paper is to reflect critically on the current relationship between hospital board oversight and patient safety. It draws on a series of interviews with key informants and policy actors in the English NHS about their perceptions about effective board oversight of safe care. In doing so the paper analyses the potential dangers and limitations of approaches to hospital board oversight which are too narrowly focused on a risk based view of organisational performance. Furthermore, it calls for engagement with theoretical perspectives that emphasise the development of trust based organisational relationships in the delivery of high quality care. These findings support the view that effective board oversight can be built on ‘trust’ characterised by organisational leadership styles and behaviours which are attentive to both staff and patient needs and concerns. In addition, effective board oversight requires the gathering and triangulation of ‘intelligence’ emanating from both national and locally generated sources. The paper concludes with a call for a re-imagination of hospital board oversight in light of these different perspectives and a look forward to an emerging research agenda in this area.

### Governance of patient safety in English hospital trusts

While successive campaigns and central directives have sought to promote and enhance quality and patient safety in the English NHS, standards of hospital care continue to be a cause for concern [3,8]. Over the past decade, the solution offered to problems over care quality has predominantly focused on developing better performance metrics and audit frameworks to help tighten external accountability and scrutiny of healthcare organisations by central government and regulatory agencies.

The prominence of external systems of accountability and control in the NHS has been highlighted by Maybin and colleagues [9] who show how hospitals are currently required to meet a myriad of nationally imposed performance targets that range across clinical and service quality standards, agreed volumes of activity, and reductions in health care-acquired infection rates. Hospitals are legally required to meet essential safety and quality requirements as part of their registration with the national healthcare regulator – the Care Quality Commission (CQC). NHS foundation trusts, those organisations defined as ‘high-performing’ hospitals and are run as not-for-profit corporations, have an additional layer of regulatory oversight provided by the economic regulator - Monitor - which imposes authorisation standards in relation to internal governance and financial viability. Two risk ratings for each NHS foundation trust are assigned in relation to: governance (traffic light red to green); and finance (rated 1-5, where 1 represents the highest risk and 5 the lowest) with monitoring and the potential need for regulatory action considered on a case-by-case basis [10].

This approach to hospital accountability has been associated with notable achievements in the English NHS in relation to improving quality and patient safety. In particular, central performance targets for infection control have coincided with reductions in hospital infection rates, especially for MRSA and clostridium difficile [11]. Notwithstanding these achievements, questions remain about the effectiveness of approaches which rely on external systems of measurement and control [12]. Brown and Calnan [13,14] summarise how such approaches to safeguarding in the English NHS based on notions of ‘risk governance’ can lead to deleterious outcomes for staff and patients as increasing rationalisation and bureaucratic controls impinge upon the more communicative, affective and intangible concerns and interactions in healthcare [15].

In England, this was recently highlighted in the accounts from patients, families and staff at the public inquiry into Mid Staffordshire hospital. Despite the self-assessment rating awarded by the Healthcare Commission (now renamed the Care Quality Commission) six months earlier as being assessed as “good”, the Inquiry highlighted how the culture of the hospital trust was not conducive to providing good quality and safe patient care. Contributory factors highlighted in the Inquiry report included styles of management based on bullying and harassment, the priority given by the board to meeting externally imposed waiting times targets, low staff morale, ongoing financial difficulties, reduction in staffing, a lack of openness about the hospital’s business, and the acceptance of poor professional standards and practice [3,4].

The findings highlighted particular failures in relation to the behaviour and conduct of the hospital board and its members [4]. The board was found to be ‘distanced from reality’ as patient safety information tended to be discussed and narrowly filtered between divisional governance groups outside of the boardroom. An emphasis on individual (medical or nurse Director) rather than collective corporate responsibility for quality and safety was identified. A lack of adequate impact or risk assessment regarding patient safety was also evident, with approaches symptomatic of a passive denial arising from a lack of effective challenge, self-criticism and engagement with the key issues. The attribution of high mortality figures to problems with coding and the creation of new governance structures as an end in itself were symptomatic of a focus on maintaining organisational processes rather than improving patient safety and outcomes.

### Bringing in the perspectives of trust and intelligence

The ongoing issues regarding effective governance in healthcare highlight the possible dangers of board oversight practices that are overly reliant on risk-based systems of assessment and monitoring. In particular, they include the dangers associated with conforming to the letter (or number) of the target at the expense of the non-targeted aspects of health care [16-19] as well as the problems associated with an over-reliance on formal quantitative performance measures at the expense of ‘soft intelligence’ which circulates through informal channels of communication such as personal and professional networks [20].

Approaches to board oversight which are focused on meeting the demands of external regulation and based on risk management also have the potential to erode the trust required to support healthcare relationships [13-15,21]. Defined as contexts or situations where social interaction is based on uncertain knowledge about the likely action of others and where one depends on their response for a beneficial outcome [21], the enactment of trust is often located in the liquid spaces between and around institutionalised roles. It can be characterised by commonly shared norms and values, the development of ethical relations which are not secured by contract or other regulatory forms, or the acknowledgment of risk in allowing oneself to be open to disappointment and regret [12,21].

Summarising Möllering [22], Brown and Calnan [14] explore how positive expectations associated with trust are made possible through common understandings of norms and values which structure the actions of the trustee. These authors point out that while trust has often been considered narrowly in terms of its impact on the truster in helping them manage uncertainty and vulnerability, it also necessarily places a moral obligation on the trustee in the light of the truster’s positive expectations, thus binding the trustee to expected patterns of behaviour [22]. In addition to the emotional component within trust (relating to the positive expectations of trusters who make themselves vulnerable), it is understood that sanctions will follow where this trust is disappointed. Here, notions of shame and embarrassment are enacted to control behaviour [23], rather than financial incentives or bureaucratic sanctions which may ultimately in themselves lead to defensive practices and stifle or inhibit ‘the social’ aspects of exchange [14].

Reflecting on the trust literature, Brown and Calnan [14] propose a form of ‘conditional trust’ as a better basis for governing healthcare professional behaviour. For these authors, trust is both reflexive (self-challenging, relational) and conditional (earned). Here the conditional element of trust can be achieved by harnessing available data to make clinical work more visible and hence accountable, but doing so in a ‘soft’ rather than an overt or coercive way. Such a view builds on the work of Davies and Mannion [24] who call for an optimal ‘balance between checking and trusting’ in relation to clinical governance. Alongside trust, checking refers to monitoring and challenging poor clinical practice. In doing so, checking provides managers with a means for holding professionals to account, particularly the potential monopoly of clinicians, to account for their actions [24].

For boards, like individuals and organisations as a whole, we argue that the neglect of trust in favour of a reliance on ‘checking’ or ‘confidence’ built through risk management systems can potentially have the effect of eroding the traditional social norms and altruistic behaviours which characterise long standing working relationships. As Smith [21] suggests, the neglect of trust in healthcare relations can lead to a disregard towards the ‘ethics of care’ in relation to the moral and affective dimensions that are involved in trusting others. Furthermore, the ‘confidence’ brought about by predictability and certainty associated with risk management may not be conducive to health and social care services which are more often characterised by the considerable uncertainty, ambiguity and daily dilemmas associated with the delivery of care [21].

When we turn to the extant literature on the nature of effective hospital board oversight of patient safety, many of these trust related themes are easily surfaced. These include effective board oversight being associated with spending sufficient time discussing patient safety issues at board meetings, reviewing and learning from patient complaints, taking time to listen to patients and staff, showing empathy to patient needs and concerns as well as clearly displaying a commitment to openness and transparency in all organisational affairs [25,26]*.* Board oversight of patient safety is also associated with having dedicated quality and safety subcommittees with the time and expertise to analyse data on the quality and safety of care, as well as being able to sustain informal relationships and dynamics between boards and organisational professional groups [27,28]. Alongside these features, the literature also points to boards obtaining intelligence gathered from information systems and pathways to produce and monitor system progress [29]. Boards that engage in reviewing and tracking organisational performance through the collection and analysis of internally generated data (quality dashboards or scorecards), national benchmarks and soft intelligence gathered from board walk rounds along with patient and staff stories and experiences are also associated with improvements in quality and safety [30-33].

These activities and characteristics of effective boards lend themselves to a wider view of board oversight that moves beyond a purely ‘risk’ based approach to oversight. In many ways, this combination of the various elements supports the view of Jennings et al. [34] that hospital boards have not only a legal duty of care and loyalty to the hospital they serve, but that they also have an ethical role in terms of the norms, expectations and values, skills, functions and competencies that they instil throughout the organisation by their behaviour. As outlined in the subsection below, Jennings et al. [34] suggest this means more than governing the hospital as an “asset” or “commodity in the market place” but also as a vibrant and viable socio-cultural system, a moral community with diverse needs, skills and contributions.

Our paper looks to build on the perspectives of trust and intelligence and analyse their utility within the context of hospital board oversight of patient safety in the English NHS. In doing so, it aims to contribute to a tradition of qualitative inquiry that looks to gain inferences from conceptual frameworks and ideas in order to broaden our understanding of these phenomena. Meyer and Lunnay [35] summarise how such an approach is akin to the generation of abductive reasoning in forming theoretical associations that enable us to discern relationships and connections that are not otherwise evident or obvious. Drawing on interviews with members of a patient safety ‘issue network’, the following sections will present a range of perspectives that analyse how board oversight of patient safety can be further developed through the nurturing of trust-based relationships and the triangulation of hard and soft intelligence gathered from both national and locally generated information sources.

### The four principles of ethical trusteeship [34]

1. Fidelity to mission: trustees should use their authority and best effort to promote the mission and keep that mission alive by interpreting its meaning over time in light of changing circumstances. The generic mission is to promote health and wellbeing, to be a civic and health resource for the community, and to be a place of respectful, well managed and competent health care provision.
2. Service to patients: Trustees should ensure that high quality is provided to patient in an effective and ethically appropriate manner. A diligent oversight of performance through participating in education and research, protecting and promoting the rights and interests of patients ensuring patients and families are partners in decision making about healthcare.
3. Service to the community: Trustees make hospitals civic institutions dedicated to improving public health and quality of civic life of the community as a whole. Hospitals are integral components of a community’s identity and traditions. Trustees do well when they bear in mind the interconnection with what occurs in the community outside.
4. Institutional stewardship: trustees should sustain and enhance the integrity of the hospital as an institution, as an effective organization for the delivery of high quality health care services and as a moral community of care giving. This translates into working with the executive management of the facility to ensure that it is well run, fiscally sound and professionally competent. They should protect the interest of all parties who rely on the hospital.

## Methods

The research examined the different ways that those involved in the ‘issue network’ surrounding patient safety within England made sense of hospital board oversight of patient safety. Our definition of an issue network originates from Heclo [36] who defines such networks as a broad collection of individuals possessing knowledge about the issue in question with some influence on policy outcomes. Such an issue network can be characterised by a wide range of interests, contacts, access, and level of agreement, resource, and power distributions among the group members [37]. Examples of these networks are often related to environmental, human rights and criminal justice issues with actors including politicians, government departments, management and policy consultants, academic researchers, and journalists.

Our research comprised 10 semi-structured interviews with stakeholders that we identified as contributing to the patient safety issue network described above. The aim of these interviews was to contribute to the initial phase of a national study of hospital Board governance of patient safety [38]. The research purposefully selected interviewees on the basis that our research required a range of stakeholder perspectives that interacted across multiple interest groups involved in shaping or attempting to shape government policy in the area of hospital board oversight of patient safety. The sample included government departments with representatives from the Department of Health, the NHS Litigation Authority, the two key regulators, Monitor and the Care Quality Commission, and a representative from the National Patient Safety Agency. We also included what could be defined as management and policy consultants with representatives drawn from the Health Foundation, NHS Confederation, as well as commentators who had contributed to the public inquiry into Mid Staffordshire Hospital Trust (see subsection below for further details). These particular individuals were selected on the grounds that they had a remit, interest and experience of quality and safety oversight at hospital board level. 4 out of the 10 participants who contributed also had previous experience of being part of a healthcare board in either an executive and non-executive capacity.

### Board oversight of quality and safety issue network sample

Our sample of interviewees incorporates a range of affiliations with actors drawn from the following perspectives:

- Health Foundation: an independent charity working to improve the quality of healthcare in the UK (n=1)
- NHS Confederation: a membership body for organisations that commission and provide NHS services aiming to bring together and speak on behalf of the whole of the NHS (n=1)
- Department of Health: ministerial department with contribution from representatives involved in patient safety policy development. (n=2)
- NHS Litigation Authority: a not-for-profit part of the NHS that manages negligence and other claims against the NHS in England (n=1)
- Monitor: an executive non-departmental public body of the Department of Health that particularly oversees and regulates Foundation Trusts (n=1)
- Care Quality Commission: an executive body of the Department of Health that regulates, inspects and reviews all adult social care services in England (n=1)
- National Patient Safety Agency (NPSA): responsible for identifying and reducing risks to patients receiving NHS care and lead on national initiatives to improve patient safety. In June 2012 the Agency became part of the NHS Commissioning Board Special Health Authority (n=1)
- Witness and seminar contributors to the Mid Staffordshire Inquiry (n=2)

The purpose of the interviews was to explore respondent views and perspectives of key dimensions of board oversight identified in the quality and safety literature. The interviews were semi structured based on a guide that asked participants about their views concerning the issues or problems facing board oversight of patient safety; the proposed solutions and suggestions for improving board oversight; the key contextual factors or conditions to achieving effective board oversight; and the recommendations these participants had for achieving effective oversight and developing boards in moving forward.

Following the tradition of patient safety studies elsewhere [39], our approach took a narratological perspective in providing an account of how individuals within this particular issue network reflected the problems and solutions related to hospital board oversight. Currie and Brown [40] note how a narratological approach is particularly valuable for the light it sheds on individual and group sense making as people interpret phenomena. Waring [39] also summarises how such storied accounts help actors give meaning to often complex and emotional situations through developing plotlines, which are sometimes ordered and linear, but more often fragmented and complex.

Data analysis looked to weave together these storied accounts into a series of narratives that connected to wider debates concerning trust and intelligence in healthcare. Here, abductive inferences were made by (re)interpreting data in conjunction with the existing theoretical and conceptual in order to reveal a more comprehensive understanding of this important aspect of patient safety governance. Discussions ensued within the research team with the emerging narratives developed and refined in an iterative process.

The NVivo software programme was used to support the coding of information that focused on particular board oversight activities that were suggested as current problems and solutions. The interviews were carried out by the lead author between October 2011 and February 2012. The National Research Ethics Service (NRES) informed us that NHS ethical approval was not required for the study. Informed consent was provided along with assurances that anonymity would be assured. In respect to the wishes of some of the interviewees quotations have been anonymised.

The elite interviewing process enabled the research to capture some of the key issues within this area of interest. That said there are notable limitations in such an approach. Elite interviewing by its very nature is limited to certain perspectives and worldviews about hospital boards and patient safety that might well exclude marginal or underrepresented perspectives. As Berry states [41], excessive personal bias and exaggerated roles represent an ongoing dynamic and challenge during the elite interview process. Certainly these findings provide us with the dominant shared narratives at the exclusion of other areas. To counter these potential issues, the researcher did look to probe the views being presented wherever possible, encouraging interviewees to think about their current opinions and perspectives, and develop particular points. For example, the emerging stories regarding the differences and dynamics of non-executives were something that became particularly apparent and warranted further probing with all the interviewees.

## Results

The results are presented in four sections that seek to capture the different perspectives put forward by interviewees. The first section examines the narratives presented by the issue network concerning current problems facing Board oversight of patient safety that relate to the limitations of risk based approaches to governance. The second section examines a collection of narratives that illustrate the challenges to effective Board oversight due to the limited knowledge and understanding of patient safety issues from individual Board members. The third section explores narratives illustrating how boards can improve their oversight through the development of trust based relationships within their organisation. Finally, the fourth section outlines an additional collection of narratives that suggest different ways that Boards can improve their oversight by paying greater attention to intelligence gathering as a means of ‘triangulating’ different versions of organisational reality.

### Board oversight as a ‘numbers game’

The perspectives from our interviews suggested that effective hospital board oversight currently faced a number of challenges. While changes in the policy agenda, particularly in response to the first Francis inquiry into the Mid Staffordshire hospital trust [4] and the Darzi review in 2008 [42], had seen quality and safety move up the political agenda, the oversight of patient safety was often compromised by the unintended consequences of relying solely on risk based governance approaches. These points attempted to illustrate the dangers of conforming to targets and quantitative performance systems at the expense of non-targeted aspects and soft intelligence [13].

In large numbers of hospitals, it was suggested that debates and discussions concerning patient safety often took second or third place and behind efforts ensure that hospital finances and central performance targets were met.

*… in most instances they do tend to defer to finance and activity. And in a crisis, especially in the financial challenges of today, it is much more comfortable for Boards to tip over in that way. Partly because of the lack of understanding, partly because in fact patient safety is incredibly broad, and partly because of the environment within which they sit…*

Within this context, the key issue facing board oversight of patient safety was the reliance on externally imposed targets, particularly those relating to infection prevention and control, as the means to assure compliance and performance. There was a view that hospital boards were heavily challenged by the regulatory environment that was designed around meeting the governance and risk based ratings set by Monitor and CQC. The targets set by these regulators had the potential to ‘dilute the message’ of patient safety for boards, by reducing conceptions of safe care into a series of national standards and priorities, or specific issues and campaigns related to hospital acquired infections such as MRSA and clostridium difficile rates.

*MRSA is one out of about a hundred things you could be doing. That’s just the one where the spotlight falls… just treating one thing in isolation, ticking those particular boxes… that is not safety; it’s not safety culture. That’s a target mentality*

*[Board] discussion centres on the numbers. So what you get is people focussed on, “Oh we’ve had two more of these”, or “Five more of those”, and the attitude of we should stop having these things… there’s so much more to it than just the numbers*

### Board oversight as a ‘silo’ activity

Alongside the problems related to Boards’ overreliance on targets and the minimisation of institutional risk, a series of narratives connected the limitations of current board oversight to the limited knowledge and understanding of patient safety issues from individual Board members. In many cases, the faith placed in external targets and assurance mechanisms was in large part connected to a lack of skills and understanding to make sense of patient safety issues and concerns. Central to this was an over reliance on performance measurement systems such as red, amber and green (RAG) ratings at the expense of critical reflection and challenge about why such issues and trends had arisen [20].

*Generally, if they’re asked to produce a list of incidents and grades of incidents, most of them can do that absolutely fine. But if they actually have to start putting together other information to cross match it, they struggle*

*the board is focused on reds… but there’s all these greens, and how do people carry on keeping them green… you learn from success as much as failings but the whole culture is on reds*

*There’s that failure to understand a moveable feast and look and see where you deviated from the norm…*

Such limited intelligence also meant that there was a tendency to ‘compartmentalise’ patient safety. Boards tended to delegate responsibility to the nurse or medical director or particular clinical directorates and subcommittee structures. While it was acknowledged such delegation was in large part a recognition by Boards that quality and safety required governance subcommittees with dedicated time and focus spent on patient safety issues, the delegation of responsibility was also problematic in that Boards were over relying on governance structures and processes as an end in themselves as opposed to connecting directly with patient care.

*I think it’s very distant for most of them. You often hear the phrase trotted out, patient safety is paramount or patient safety is the most important thing to us, but in terms of what’s actually done and delivered, that doesn’t really seem to be backed up… They may wish to be assured that someone in the organisation, as it were, deals with patient safety, however, I don’t know the extent to which they actually realise the role that they themselves have to play in it…*

Such limited knowledge and understanding of patient safety among board members, particularly non-executives, also meant they were often inhibited in challenging and posing critical questions about safety issues and concerns. This was exemplified by non-executive directors (NEDs) who tended not to have a clinical or operational background in healthcare but have skills and expertise in relation to finance, industry and legal settings.

*the non-execs in the main feel inhibited to challenge in relation to patient safety and are fearful of what the right questions are… especially when it comes to clinicians… [NEDs] who are recruited tend not to be from a clinical background… the fact that they don’t have a clinical background I personally think inhibits their ability to ask the right questions in relation to safety*

This later point reflects a view of Boards unable to or lacking confidence in the ability to self-challenge. Board dynamics were leading to defensive practices as certain Board members would ‘hide’ from discussions and dialogue related to patient safety matters rather than create contexts and situations for social interactions related to patient safety issues and concerns [21].

### Board oversight as building trust relationships

In light of the challenges facing boards, our interviewees put forward a number of suggestions about how board oversight of patient safety could be improved. At the centre of these proposals was the view that oversight needed to become ‘value driven’ in the development of relationships that were more sensitive and responsive to organisational needs and concerns. Rather than a ‘tick box’ focus on meeting particular targets, board oversight concerned building trust through the development of better social interactions and interpersonal connectedness with their organisations [21].

Particular board activities and practices were put forward by the interviewees as effective strategies for enhancing trust in organisational relationships These included providing a greater amount of time and emphasis on board discussions and dialogue about patient safety, developing enhanced leadership skills that allow patient safety to be more visible, and seeing board members as ‘stewards’ with responsibility for developing a culture of partnership within the organisation.

*go past a building site these days and you’ll see no hat, no shoes, no entry. Or you’ll see how many days since the last safety incident. You may well see a visible manifestation of safety on that site is important. What do you see when you go into most NHS institutions? There isn’t much manifestation of a visible safety culture. That’s something that the board could set. They could lead. They could set the example. They could support…*

*Stewardship is the right thing, around setting the culture, asking the right questions. I think they need courage… to say we’ve got this issue, we don’t know how to fix it, but what we’re going to try and do is work out a way together of finding solutions...... in the plane, whoever says I can hear a noise on the engine, you all stop and listen. You don’t beat them up for raising something and they’re beneath contempt. And that culture of safety is a million miles away until we’ve got everybody feeling empowered, respected and encouraged…*

Leadership styles were mentioned with regard to the way board members needed to ‘set the tone’ for the organisation in enhancing the quality of care. To gain a deeper understanding of the issues, interviewees described board members displaying characteristics that centred on a curiosity to ask questions and be open to critical reflection about ‘uncomfortable truths’ within the organisation. Such views supported the literature in this area which relates those boards who discuss, review and learn from patient safety events with higher performing organisations [25,27,30].

*in the end I think actually it comes down to something like authenticity… it’s about listening to people and it’s about being genuinely interested in their concerns and it’s about doing something about it… authenticity comes from people owning the problem and feeling motivated to do something about it*

*it’s about setting some very clear parameters from the board, some very clear messages, some quite tough objectives, but they need to be quite realistic even if they are tough*

The role of the chief executive was thought to be important here in setting the tone for the behaviours and values of patient safety. Patient safety needed to be ‘a personal cause’ as was engaging staff and patients about their concerns.

*where the chief exec can be so important is where they say or they’re attempting to steer or take a trust down a certain route from a patient safety perspective and that they don’t just say that… they enable by giving resources to make it happen, they provide a personal leadership commitment by being involved, turning up in person – all the things that demonstrate personal commitment that turn something from being a paper exercise to something that’s real and visible on the ground and happening*

It was suggested that the role of the chair was central to allowing open discussion at board meetings by encouraging members to raise salient issues. The medical and nurse directors acting as custodians for clinical governance also had a role to play in setting the patient safety culture of the organisation as were finance directors in getting involved in patient safety initiatives and concerns.

*The board have got to set the tone for behaviours and values, that that’s not to be tolerated. It has to come from the top and it would have to come from the Director of Nursing who’s got to get out of her or his office…They’ve got to challenge their executives to know how do you know what’s happening on the wards? And if you don’t know, why don’t you know, because we want to know?*

*raising awareness amongst the finance directors of what they could do that would really make a difference to patient care, because… whether they themselves understand that personally can make a difference… in particular in an understanding of business cases around patient safety, I think that could be hugely powerful.*

Non-executive directors were also important here in being able to actively challenge executive decisions and hold the board to account. It was suggested by some that boards needed greater input from non-executives in providing the necessary critical reflection in ‘holding up a mirror’ to the organisation by asking critical and probing questions which executive members would need to answer.

*non-exec directors should be demanding… demanding to see real commitment, to see initiatives, to really see culture change and quite often to lead it as well.*

*I think you sometimes need to challenge what you’re seeing because if you’re getting people coming through time after time and saying, ‘Oh, I’m so delighted this has happened.’ You think, hang on....*

An additional suggestion for fostering greater inter-connectedness with the wider organisation was opening up board business to greater staff, patient and public scrutiny. This it was felt had the potential not only to further hold the board to account but also to create further connections and interactions with the organisation and the community that the hospital served.

*I think maybe Boards have gone a bit too far in terms of not having people that are representative of real people on there, and I don’t mean it disparagingly, but a lot of finance, a lot of accounting people, a lot of business people… Someone with their heart in the community would be quite nice or an adversary would be a nice thing. So I think there’s something about the makeup of Boards which needs to reflect a bit more the purpose and the purpose of patients and patient care.*

These suggestions to improve the connection between boards, patients and staff point to the potential of trust to engender positive organisational norms and values. The institutional spaces between and around executive director roles needed to be filled through relationships, interactions and values rather than necessarily secured or undermined by formal contracts and external performance targets.

### Board oversight as gathering intelligence

Interviewees, particularly those from CQC and Monitor, suggested that there remained an important role for independent regulation and inspection. This was reinforced by the view that the introduction of external systems of performance measurement and monitoring had yielded improvements in several areas, most notably in relation to reductions in hospital acquired infection rates.

Nevertheless, our interviewees raised a number of points about the over reliance on formal quantitative performance measures in relation patient safety which came at the expense of ignoring the potential insights gleaned through soft intelligence networks. Boards in this respect needed to be exposed to different types of ‘hard’ and ‘soft’ information concerning patient safety in order to increase their intelligence about the issues at hand.

In relation to hard performance data, it was felt that greater efforts needed to be made to disaggregate information about particular trends within the organisation rather than rely solely on an aggregated picture of the organisation as red, amber or green ratings. For example, one interviewee suggested that the Quality Risk Profiles (QRPs) capturing mortality data, re-admission data, and outlier data combined with data on patient outcomes in relation to dignity, nutrition and privacy provided a useful basis for hospital boards to build on. In addition, the promotion of ‘soft’ information was called for with some interviewees suggesting that boards should supplement scorecards and metric based approaches with a narrative or ‘reality check’ that connected with patient and staff stories about particular experiences in the board room [25-27].

*if patients are saying there’s a worry, if staff are saying things aren’t great and if the performance is beginning to go down on some of the things that trusts had done well in the past, those three things feel like a place to start having other conversations. Then I suppose it’s what you put in those baskets of indicators in those three places*

*I completely buy into dashboards… But what is that actually saying? And what’s the narrative to go with that that persuades the public that you’ve recognised something, and you’re doing something about it, and you are minimising risk and harm as a consequence? I think the dashboards are one thing but the second thing is what the patient experience in this is? What is the patient understanding of this? And what’s the patient’s story to go with it?*

These findings were also linked to ideas suggesting that enhancing the intelligence available to the Board about hospital performance could be gained by individual members proactively seeking to ‘triangulate’ hard performance data with different information sources. A more proactive stance in relation to soft intelligence was needed with Board members being more open to informal feedback and conversations with patients and staff regarding the quality of care:

*Whether you have staff who will come in as observers to the board and you’ll say to them, is that how it feels like on your ward, as well as you’re getting out and about and talking to people and bringing that intelligence back to the board. I think that is crucial because … you could get into a little bubble where it all looked lovely because you’ve got mostly greens and good answers for the ambers and the reds. So I do think that reality check is crucial*

*storytelling needs to become much more a part of the conversation quite literally and how we communicate these issues*

A number of additional benefits could also be gained from the accumulation of soft intelligence. They included providing Board members with improved confidence when faced with any uncertainties and ambiguities identified in performance data. Soft intelligence could also facilitate improved dialogue between Board members as the exposure to such information could facilitate the sharing of experiences and insights about patient safety issues and concerns.

## Discussion

The narratives presented above highlight how effective hospital board oversight of quality and safety can be related to a variety of governance activities. They draw our attention to the limits of risk based governance and provide further evidence to arguments raised elsewhere about the dysfunctions associated with overly excessive forms of risk management [13]. In particular, the attempts to reduce risk and manage uncertainty through a focus on performance targets and measures often fail to account for the wider experiential outcomes of organisational life [15]. While there is some support for dedicated time and resources spent on quality and safety issues within subcommittee governance structures, care is needed to ensure that the promotion of governance structures do not reinforce or legitimate a silo view of patient safety governance where the oversight of safety issues lies solely within the directorate of either the nurse director or the medical director, deflecting the corporate responsibility from the board as a whole.

Alongside these apparent challenges, the narratives also direct us to different ways board oversight functions and duties could be improved. The suggestions made calling for Boards to further develop organisational relationships and values, as well as display leadership that embodies patient safety appear suggest that a change in current approaches to Board oversight is required. The notion of trust emerges here in the recommendations that Boards do more to develop commonly shared norms and values that go beyond regulatory forms into the realm of normative and communicative frameworks that encourage desired patterns of behaviour [22]. These findings also go to the very heart of what Jennings et al. [34] see as hospital governance that is more than a “commodity” but also involves the creation of a vibrant and viable hospital system with diverse needs, skills and contributions.

For many hospital organisations, the combination of these types of behaviours and practices may already be in existence. However, there is still work to be done in addressing the issues we raise. Within the English NHS and beyond, hospital organisations are being required more than ever to respond to quality and safety concerns and demonstrate that they deliver compassionate care through the promotion of relationships built on empathy, respect and dignity [26,43,44]. The building and strengthening of board skills to lead such changes represent an important case in point leadership development programmes are being put forward as a means to develop the necessary skills in relation to involving, communicating and engaging staff, patients and users and other stakeholders [43]. Strengthening board recruitment is also being proposed with an explicit focus on values, attitudes and behaviours of potential candidates in relation to care, compassion and respect [26]. This is particularly interesting in light of recent findings suggesting that having more Board members with clinical backgrounds is often associated with improved organisational performance [45].

The narratives provide a number of insights into the trust dynamics that are associated with effective board oversight of quality and safety. For example, they provide insights into how staff, patients and the public can act as truster and board as trustee with calls for a greater presence of staff, patients and the public within Board meetings in order to shape strategy and hold the executive to account. They also highlight elements of the alternative dynamic with boards acting as truster and organisational staff as trustee through the engagement and dialogue with performance information. This latter form of trust draws attention to how boards as truster need to gather and ‘triangulate’ intelligence through hard performance data such as HCAI rates but also soft forms of information obtained through feedback and interactions with staff, patients and the public. This form of trust requires Board members to go beyond standard performance metrics and proactively engage in the qualitative dimensions of hospital life embodied in the stories, experiences and emotions related to patient care.

While these findings highlight a number of ways to achieve different trust relationships, there are some important elements of the trust relationship that warrant further analysis. Our findings support the points made by Brown and Calnan who describe how trust is achieved by placing moral and emotional obligations on the trustee in the light of the truster’s positive expectations. Yet our findings remain largely underdeveloped in thinking through how trust is achieved when it is understood that sanctions will follow where this trust is disappointed i.e. the role of sanctions to control behaviour. Building on others [24], Brown and Calnan [14] develop this line of inquiry as a form of ‘conditional trust’ for governing healthcare professional behaviour achieved principally by making clinical work more visible and accountable, but doing so in a ‘soft’ rather than an overt or coercive way. Their suggestions for achieving this form of trust include the creation of outcomes evidence not accompanied by external monitoring; the encouragement of clinical leadership to oversee and coordinate governance systems; and clinically owned criteria to assess performance and refine standards.

While it has been beyond the scope of these narratives, further study of what such effective sanctions would look like for hospitals and how boards can enact these sanctions within trust relationships is needed. One such line of inquiry in relation to Board oversight of patient safety would be to focus on the Board relationships and interactions with whistleblowing. Whistleblowing – the disclosure, either to a person in authority or in public, of information concerning unsafe, unethical or illegal practices – would provide a fruitful line of inquiry in this regard. How Boards interact with and encourage staff to raise concerns is particularly relevant, in the context of the current focus on improving whistleblowing policies in the NHS [46]. Additional lines of inquiry can also be identified in the study of how Boards interact with increasingly personalised performance measures across clinical specialities. This is particularly relevant within the context of recent developments in the English NHS to publish data on patient outcomes for 5,000 NHS surgeons [47].

## Conclusion

Effective hospital board oversight of quality and safety requires greater engagement with issues related to trust and intelligence. We suggest that further research is required to develop our emerging analysis. In particular, research that looks at how the theoretical perspectives of trust and intelligence translate throughout healthcare organisations and across different healthcare governance arrangements would undoubtedly develop these perspectives. In terms of methodological development, our research goes some way to developing and refining the application of theoretical perspectives of trust and intelligence in the context of hospital board governance. However, further work is needed to tease out these perspectives in particular healthcare contexts, and in particular how these are framed by different groups and audiences. Questions concerning what make trust and intelligence possible, for whom and in what circumstances is needed. Furthermore, the contextual conditions required for trust and intelligence to occur and be sustained is something that future research will be required to address.
